# Discriminant Analysis of Main Prognostic Factors Associated with Hemodynamically Significant PDA: Apgar Score, Silverman–Anderson Score, and NT-Pro-BNP Level

**DOI:** 10.3390/jcm10163729

**Published:** 2021-08-22

**Authors:** Anna V. Permyakova, Artem Porodikov, Alex G. Kuchumov, Alexey Biyanov, Vagram Arutunyan, Evgeniy G. Furman, Yuriy S. Sinelnkov

**Affiliations:** 1 Department of Pediatric Infectious Diseases, Perm State Medical University, 614990 Perm, Russia; derucheva@mail.ru; 2 Federal Center of Cardiovascular Surgery, 614990 Perm, Russia; porodickov.a@yandex.ru (A.P.); a.bianov@yandex.ru (A.B.); cvsvagr@mail.ru (V.A.); fccvs@permheart.ru (Y.S.S.); 3 Department of Computational Mathematics, Mechanics, and Biomechanics, Perm National Research Polytechnic University, 614990 Perm, Russia; 4 Department of Pediatrics, Perm State Medical University, 614990 Perm, Russia; 5 Department of the Intermediate Level and Hospital Pediatrics, Perm State Medical University, 614990 Perm, Russia; furman1@yandex.ru

**Keywords:** premature newborns, hemodynamically significant patent ductus arteriosus, Apgar score, Silverman–Anderson score, NT-pro-BNP

## Abstract

Hemodynamically significant patent ductus arteriosus (hsPDA) in premature newborns is associated with a risk of PDA-related morbidities. Classification into risk groups may have a clinical utility in cases of suspected hsPDA to decrease the need for echocardiograms and unnecessary treatment. This prospective observational study included 99 premature newborns with extremely low body weight, who had an echocardiogram performed within the first three days of life. Discriminant analysis was utilized to find the best combination of prognostic factors for evaluation of hsPDA. We used binary logistic regression analysis to predict the relationship between parameters and hsPDA. The cohort’s mean and standard deviation gestational age was 27.6 ± 2.55 weeks, the mean birth weight was 1015 ± 274 g. Forty-six (46.4%) infants had a PDA with a mean diameter of 2.78 mm. Median NT-pro-BNP levels were 17,600 pg/mL for infants with a PDA and 2773 pg/mL in the non-hsPDA group. The combination of prognostic factors of hsPDA in newborns of extremely low body weight on the third day of life was determined: NT-pro-BNP, Apgar score, Silverman–Anderson score (Se = 82%, Sp = 88%). A cut-off value of NT-pro-BNP of more than 8500 pg/mL can predict hsPDA (Se = 84%, Sp = 86%).

## 1. Introduction

Patent ductus arteriosus (PDA) is an extra blood vessel found in babies before birth and just after birth. In full-term newborns, it usually closes shortly after birth, and in premature infants it functions in most cases for up to one month [1]. Long-term persistence of the duct contributes to its hemodynamic significance (hs), when the increasing hypoperfusion of the brain and internal organs causes the development of serious long-term outcomes (such as bronchopulmonary dysplasia, intraventricular haemorrhage, necrotizing enterocolitis [2,3], and chronic lung disease [4,5]) associated with an increase in mortality [6].

The current therapeutic approach in the form of prescribing preterm infants non-steroidal anti-inflammatory drugs (COX inhibitors) in order to close the PDA earlier is largely subjective and is associated with side effects, when the harm from treatment may outweigh the benefits [7], since PDA can close spontaneously by 44 weeks of postnatal age or later [8]. As Benitz et al. [9] reported, “The ductus is likely to close without treatment in infants born at >28 weeks’ gestation (73%) [10] and in those with birth weight >1000 g (94%) [11]”.

Surgical PDA ligation is also significantly associated with a high likelihood of adverse outcomes and has no long-term benefits [12,13].

Contemporary tactics for managing patients with PDA are observational in nature. The decision on therapy is made in the case of hemodynamic significance of PDA. According to the American Academy of Pediatrics, a combination of standard echocardiographic assessments of PDA and biomarkers should be preferred [11,12].

It should be noted that there are several approaches to define hsPDA [14]. The hemodynamic effects of the PDA using clinical signs, echocardiographic parameters, and other objective assessments can be used to declare a PDA as hemodynamically significant [15].

Brain natriuretic peptide and its inactive fragment N-terminal pro-BNP (NT-pro-BNP) are reliable markers of ventricular dysfunction in adults and children [16]. It was noticed that BNP and NT-pro-BNP are similarly useful for assessing PDA size in preterm infants [17]. Serial BNP measurement is also valuable for monitoring treatment response [18]; however, whether plasma BNP has value as a marker for predicting treatment response to COX inhibitors remains unclear.

Therefore, timely prediction of the hemodynamic significance of PDA in premature infants is especially important in the early neonatal period (during the first 48–72 h), and contributes to the timely appointment of adequate therapy [19,20].

Recently, BNP estimation was shown to be a useful prognostic marker of all-cause mortality in extremely low body weight infants with bronchopulmonary dysplasia-associated pulmonary hypertension [21]. All currently known methods of PDA closure are associated with adverse effects, when the treatment harms may outweigh benefits [7]. One of the ways to solve the problem is to develop prediction models to permit early identification probabilities of persistent PDA [22]. Studies in preterm infants highlighted echocardiographic [23], biochemical [24], and clinical markers [8,25] correlating with hemodynamic significance of a patent ductus arteriosus. It is believed that prognostic factor combinations provide better risk assessment than separate markers [26]. Our study included 40 different clinical, laboratory, instrumental, and anamnestic criteria, including assessments according to the standard Apgar and Silverman–Anderson scores.

Thus, the hypothesis of the present study was to establish a combination of clinical predictors with an acceptable degree of specificity and sensitivity predicting hsPDA in preterm infants on day 3 of life. To solve the problem, discriminant analysis was applied.

## 2. Materials and Methods

### 2.1. Subjects and Data Collection

**Figure 1:**
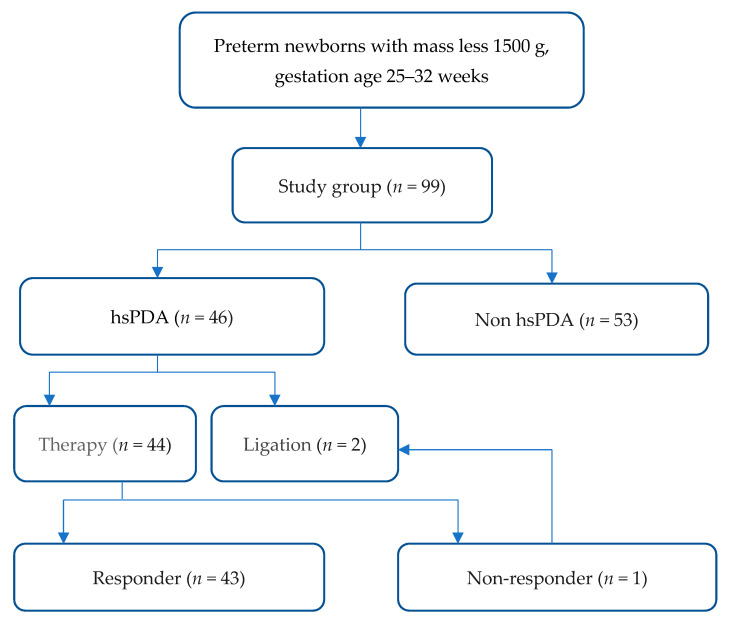
Study flow chart.

A retrospective study was carried out in Perm Krai Perinatal Center. Data on 99 infants born from October of 2018 to April of 2020 were collected. Patients with gestational age ranging from 25 to 32 weeks and birth weights ranging from 500 up to 1500 g were included in the study (Figure 1). Newborns with developmental defects (including combined congenital heart defects), infectious diseases, or severe somatic disorders were excluded from the study. The Apgar score assessed the state of the newborn child at the fifth minute of life. To identify current or threatening respiratory distress syndrome during the first hour of life, an assessment of the respiratory severity score (RSS) designed by Silverman and Anderson in 1956 to quantify respiratory distress among neonates was used [27]. Hedstrom et al. [28] pointed out that this clinical scoring system would be correlated to other laboratory parameters by which respiratory distress is evaluated in high-resource settings to prognosticate a patient’s respiratory trajectory.

NT-pro-BNP level in plasma was evaluated on the third day of life by reagent set (JSC VECTOR-BEST, Novosibirsk, Russia). Moreover, echocardiogram initial screening to visualize PDA was performed on the third day of life.

The definition of an hsPDA was based on previously established criteria [14,29,30] and was defined by the presence of the following three factors: narrowest ductal diameter >1.5 mm; LA/AO ratio of more than 1.4:1; retrograde descending aortic flow more than or equal to 50% of antegrade flow.

A Vivid Q cardiovascular ultrasound system (General Electric, Boston, MA, USA) was utilized during the study.

Clinical, demographic, and biochemical data were obtained retrospectively from medical records.

Written informed consent was obtained from patients’ parents. The study was approved by the Ethics Committee of S.G. Sukhanov Cardiovascular Center, Perm, Russia.

### 2.2. Data Analysis

In the case of describing quantitative indicators with a normal distribution, the data obtained were combined into variation series, where the arithmetic mean values (M), standard deviations (SD), and confidence limits (a 95% confidence level) were calculated. Comparisons were performed using a Student’s *t*-test. Aggregates of quantitative indicators, the distribution of which differed from normal, were described using the values of the median (Me) and the lower and upper quartiles (Q1–Q3) and compared by the Mann–Whitney test. The nominal data were described with the indication of absolute values and percentages. Comparison of nominal data was carried out using a Pearson χ^2^ test.

BNP and NT-pro-BNP were log-transformed to obtain a normal distribution for analysis. The correlation coefficients were obtained to describe the relationship between BNP and NT-pro-BNP and the presence and size of a PDA. A *p*-level of <0.05 was considered significant.

To create a predictive model, the discriminant analysis method was used. The PDA hemodynamical significance indicator was defined as a dependent variable, taking two values, which were coded as 1 (yes) and 0 (no), respectively. Quantitative indicators were used as independent variables.

In total, 40 variables were used for discriminant analysis: anthropometric indicators (weight and height at birth), gestational age, clinical assessment of the cardiovascular and respiratory systems at birth (Apgar score at 1, 5, and 10 min of life, respiratory severity score by Silverman–Anderson method during the first hour of life), baby therapy after birth (surfactant, inotropes), type of respiratory support (DUOPAP, NCPAP, mechanical ventilation), results of neurosonography and radiography, laboratory data (hemoglobin, platelets, NT-pro-BNP), echocardiographic criteria, maternal history (the number of pregnancies, births, abortions), obstetric history, and somatic diseases of the mother. The groups of variables for analysis are provided in Table S1 (see Supplementary material).

The model was created on the principle of the possibility of predicting the dependent variable based on the values of the measured factor signs and was presented in the form of the following equation:

y = a_0_ + a_1_x_1_ + a_2_x_2_ + … + a_n_x_n_, (1)

where y is a dependent variable, а_0_ is a constant, а_1…n_ are regression coefficients, x_1…n_ are independent variables (factor attribute values). The stepwise method selected the discriminant variables on basis of Wilks’ lambda statistic, and in general, the F value was set at F Entry = 3.84 and F Removal = 2.71. Assuming that the mean discriminant score of the controls was ${\bar{Z}}_{а}$, ${\bar{Z}}_{b}$ for the cases and $\bar{Z}$ for the total, then $\bar{Z}$ = $({\bar{Z}}_{а}$+$\;{\bar{Z}}_{b}$)/2. According to the discriminant function, we calculated the discriminant score of $Z_{i}$ for each subject; if $Z_{i}\;$>$\;\bar{Z}$, the subject is considered highly likely to be a case, and if $Z_{i\;}$≤$\bar{\;Z}$, the subject is regarded as a control. The diagnostic efficiency of the model was defined as the proportion of correctly predicted values with respect to the total number of analyzed observations.

We used binary logistic regression analysis to predict the relationships between NT-pro-BNP and hsPDA. The predictive model can be written as follows:

(2) $$P=\frac{1}{1+e^{-z}}$$

z = a_0_ + a_1_x_1_ + a_2_x_2_ + … + a_n_x_n_, (3)

where *P* is the probability of hsPDA, x_1_...x_n_ are risk factor values, a_1_...a_n_ are regression coefficients. The selection of independent variables was carried out by the step-by-step direct selection method using Wald statistics as an exclusion criterion. The statistical significance of the resulting model was determined using the χ^2^ criterion. To assess the diagnostic significance of quantitative signs in predicting the outcome, calculated using a regression model, the method of analysis of ROC curves was used. The relationships were graphically represented by probability curves. Receiver operator characteristic (ROC) analysis was used to determine the area under the curve (AUC). The highest value of the Youden index was used to determine the optimal cut-off point. Sensitivity, specificity, positive predictive values (PPV), negative predictive values (NPV), and positive and negative likelihood ratios (LR+ and LR−) were calculated. Statistical analysis was performed using IBM SPSS Statistics v.26 software. 

## 3. Results

### 3.1. Baseline Characteristics

The clinical characteristics of patients are shown in Table 1. According to the definition of echo criteria for the significance of a functioning ductus arteriosus, the following data were obtained: the duct is significant in 46 patients (46.4%) and not significant in 53 (53.6%). Only 26 (26.3%) babies were born naturally; the rest were born operatively (cesarean section). In the hsPDA group, vaginal delivery was more often, 76.1% (35/46) compared to 32.4% in the non-hsPDA group (23/54), OR = 4.3 (95% CI 1.8–10.2). Threat of miscarriage as an indicator in infants with hsPDA is noticed in 74% (34/46) of cases and in 39.6% (21/53) of cases in the non-hsPDA group (*р* = 0.001), OR = 3.46 (95% CI 1.3–9.15). A total of 45 (45.4%) mothers received antenatal corticosteroid therapy: 41.3% (19/46) in the hsPDA group, 49.0% (26/53) in the non-hsPDA group, OR = 0.73 (95% CI 0.3–1.6). Respiratory support was required in 91.3% (42/46) and 84.9% (45/53), OR = 1.8 (95% CI 0.5–6.6) in the hsPDA group and in the non-hsPDA group, respectively. An Apgar score 5 min after birth ≤6 points was found in 26.1% (12/46) in the hsPDA group, 9.4% (5/53) in the non-hsPDA group, OR = 3.4 (95% CI 1.1–10.5). A Silverman–Anderson score (respiratory severity score) [27] of respiratory distress over 5 was found in 71.7% (33/46) and 49.1% (26/53), OR = 2.6 (95% CI 4–6.1), in the hsPDA group and non-hsPDA group, respectively (see Table 1).

Echocardiographic evaluation of PDA in the preterm infants was assessed on the third day of life. The average (SD) diameter of the PDA in children with hsPDA is 2.78 ± 0.72 mm, in children with non-hsPDA—1.26 ± 0.66 mm (*p* = 0.001). Pulmonary hyperperfusion was assessed by an LA/Ao ratio ≥1.5, which indicates a heavy load on the left side of the heart: in the hsPDA group, 73.9% (34/46), with non-hsPDA—3.8% (2/53), OR = 72.3 (95% CI 15.2–343). Systemic hypoperfusion was assessed by the presence of retrograde blood flow equal to or greater than 50% of direct blood flow in the descending aorta: in the hsPDA group, 76.1% (35/46), in children with non-hsPDA—7.5% (4/53), OR = 39 (95% CI 11.5–133) (Table 2).

**Figure 2:**
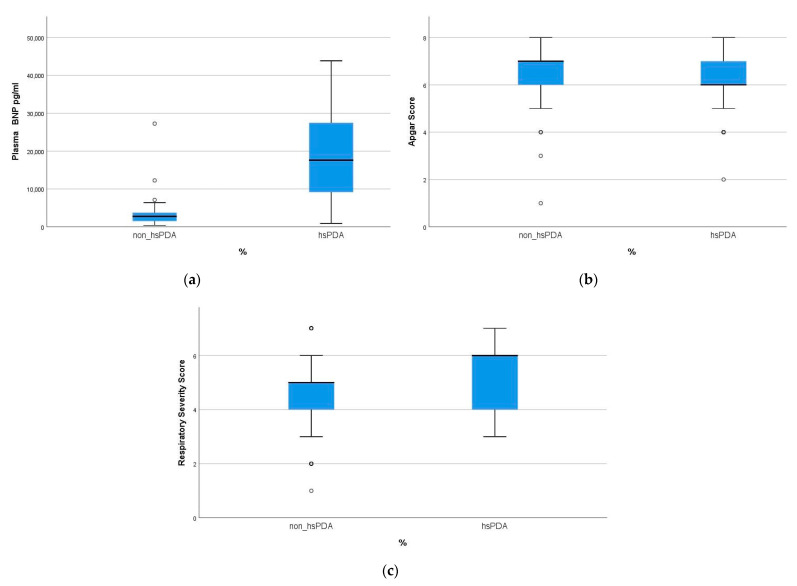
Main factors affecting hsPDA. (**a**) Plasma BNP values in the groups with an hsPDA and non-hsPDA; (**b**) Apgar score; (**c**) Silverman–Anderson score. The lower and upper bounds of the boxes indicate the 25th and 75th percentile values, respectively. The horizontal lines indicate the median and the whisker bars represent the 10th and 90th percentiles.

BNP levels in the hsPDA group were significantly higher: the median value (IQR) of BNP was 17,600 (Q1–Q3: 9172–27,237) pg/mL, and 2773 (Q1–Q3: 1532–3739) pg/mL in the non-hsPDA group (*p* = 0.001). Figure 2 contains values of BNP in plasma, Apgar score, and Silverman–Anderson score in patients with hsPDA and non-hsPDA.

**Figure 3:**
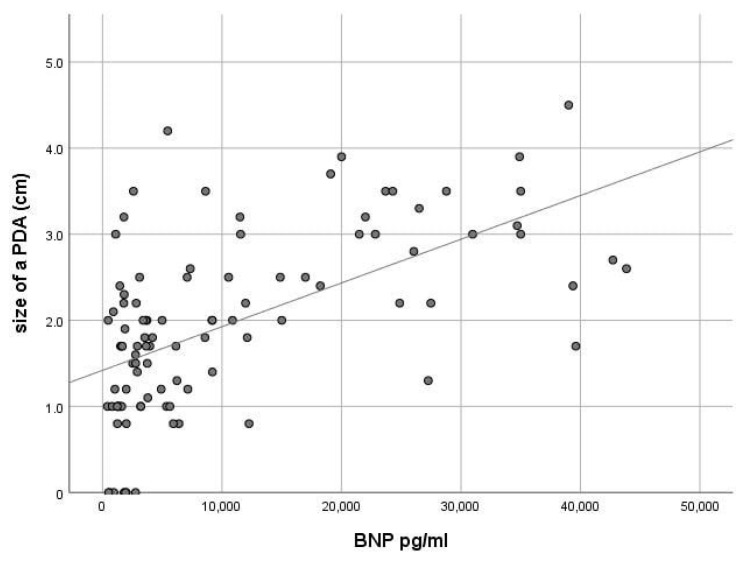
Correlation of BNP and PDA size. R = 0.60, *p* = 0.001.

There was a direct correlation between the NT-pro-BNP values and hsPDA echo criteria: for PDA r = 0.60, *p* = 0.01 (95% CI 0.31–0.68), determination coefficient r2 = 0.36 (Figure 3), for retrograde blood flow in the postductal aorta r = 0.51, *p* = 0.05 (95% CI 0.26–0.66), r2 = 0.26, for the left atrium/aorta LA/Ao ratio r = 0.51, *p* = 0.05 (95% CI 0.34–0.70), r2 = 0.26. Correlation analysis showed no dependence of NT-pro-BNP level on gestational age (r = −0.09, *p* = 0.343) and body weight (r = −0.10, *p* = 0.316).

### 3.2. Prediction Model

Using the results of the univariate logistic regression analysis, a risk prediction model of hsPDA was constructed by a stepwise Fisher discriminant analysis (F Entry = 3.84, F Removal = 2.71) based on the three screened variables that were statistically significant. The stepwise discriminant analysis showed that Wilks’ lambda, as a test of the discriminant function, was significant (lambda = 0.48, chi-square = 70.0, df = 3, *p* < 0.001), and three variables were selected, as follows: Apgar score ($X_{1}$), Silverman–Anderson score ($X_{2}$), and lg BNP (pcg/mL) ($X_{3}$).

The final standardized discriminant function was computed as follows:

*Z* = −10.2 − 0.039 × *Х*_1_ + 0.277 × Х_2_ + 2.433 × *Х*_3_. (4)

In the discriminant analysis, *Z*_non-hsPDA_ = −0.961, *Z*_hsPDA_ = 1.107, and *Z* = (0.852 − 0.823)/2 = 0.073. Then, we calculated the discriminant function value of *Z* for each subject; if *Z* > 0.073, the subject was considered highly likely to be a case of hsPDA, and if *Z* ≤ 0.073, the subject was regarded as normal. The classification results obtained using the discriminant function are presented in Table 3. The rates of correct prediction were 82.6% for the hsPDA cases (sensitivity) and 88.7% for the controls (specificity), and the positive and negative predictive values were 84.0% and 86.0%, respectively. After that, cross-validation, which gives a more “optimistic” forecast than the conventional validation method, was performed. Thus, as a result of cross-validation, we a obtained sensitivity value of 85.9%, which determines the applicability of the obtained classification in practice.

**Figure 4:**
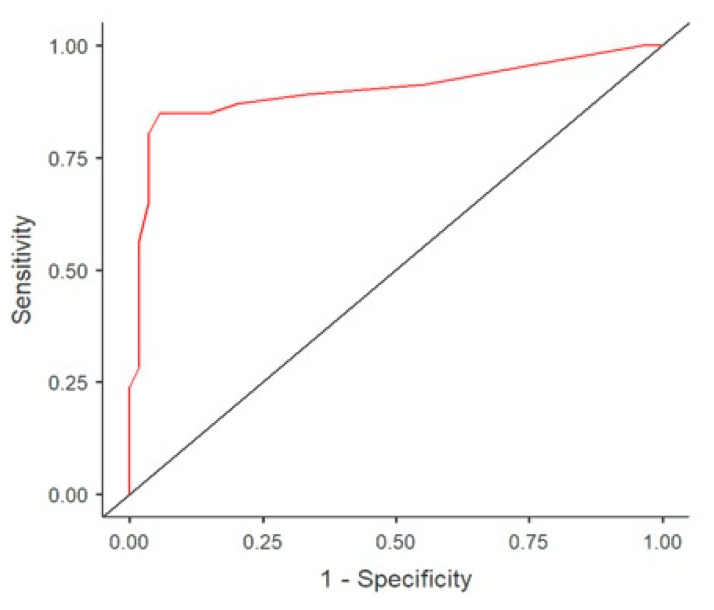
Receiver operating characteristic curve demonstrating that a NT-pro-BNP level of 8500 pg/mL results in a sensitivity of 94.2% and a specificity of 83.7% for predicting hsPDA.

Analysis using a receiver operating characteristic curve shown in Figure 4 identified a peak NT-pro-BNP cut-off value of 8500 pg/mL to have the best combination of sensitivity (83.7%) and specificity (94.2%) for predicting hsPDA. ROC curves were generated for various NT-pro-BNP levels in the diagnosis of hsPDA. The area under the curve was maximum (0.89, 95% CI 0.83 to 0.91) at a cut-off value of 8500 pg/mL. The positive predictive value at this cut-off was 94% while the negative predictive value was 85%. The likelihood ratio for a positive test was 14 and for a negative test it was 0.2.

## 4. Discussion

Early diagnosis of hsPDA in preterm infants is undoubtedly a clinical problem as it is associated with serious clinical outcomes and in-hospital mortality. However, over the past decade, there has been a tendency towards a decrease in the use of both medical and surgical treatment of hsPDA in this group of patients [31].

We analyzed the statistical data of the Perm Krai Perinatal Center in the period from 2015 to 2019, according to which the proportion of children who received surgical PDA ligation decreased 3.7 times (from 17.0% to 4.5%). The median age of the operated children was 20 months. Of the patients participating in our study, two children (aged 32 and 43 days) underwent surgery.

In our practice, we adhere to the conservative approach aimed at improving the effects of left-to-right shunting by fluid restriction, diuretics prescription, etc. Good results of conservative treatment without surgery have been reported in a number of studies, according to which the frequency of spontaneous closure of PDA at 36 weeks postmenstrual age reaches 83–85% [8,32,33].

However, at present, there is a problem of lack of evidence in favor of using a conservative approach to the treatment of premature infants with PDA, since the currently existing echocardiography-guided approach to PDA management is characterized by significant variability [29]. Ultrasound, although accepted as the gold standard, has a number of limitations, as it is expensive, requires interpretation by a cardiologist, can cause stress in such fragile and immature newborns, and may not be available in resource-limited settings.

Echo criteria traditionally used for hsPDA diagnosis, such as PDA diameter, for example, do not allow prediction of serious clinical outcomes [34,35]. For this reason, a great importance is attached to predictive models that take into account clinical, laboratory, and instrumental data [36,37,38].

For example, Kindler et al. [39] developed a short clinical score with number of signs in addition to conventional echocardiography criteria for prediction of hsPDA. The signs used in the study were pulsations of the precordium, heart rate, apnea or mechanical ventilation, femoral pulses, systolic murmur, hepatomegaly, acidosis, and pulmonary deterioration. In our study, respiratory failure was assessed during the first hour of life with the Silverman–Anderson score. We hypothesized that newborns with high RSS (≥5) have higher carbon dioxide partial pressure (PCO_2_) values, as a result of which there is a decrease in muscle wall tone, leading to the duct staying open. The advantages of applying the Silverman–Anderson scale include simplicity and the possibility of use in conditions with limited resources. In our study, we initially used 40 diagnostic criteria, but the greatest ability in predicting probabilities of persistent PDA was established for a combination of criteria: Apgar score ≤ 6 points, Silverman–Anderson score ≥ 5 points, and NT-pro-BNP over lg3.9 pg/mL.

The novel contribution of our work is that our suggested combination of predictors is new, having not been previously described, and is substantiated by the results of discriminant and variance analyses. We have identified the combination of predictors that has high specificity values (88.7%, 95% CI 0.77–0.94), leading to a low probability of a false positive diagnosis, which is of practical importance for making a clinical decision on surgical correction of the duct.

As for the clinical utility of measuring the biomarker NT-pro-BNP for prediction of hsPDA, the increase in its plasma concentration is significantly correlated with the increase in the left atrium arising from PDA [40]. The biomarker NT-pro-BNP in preterm infants is described by many authors both for diagnosis and for the initiation of medical or surgical treatment of hsPDA [41]. We found one systematic review that included 11 studies that differed in methodological quality, age at testing, gestational age, type of commercial assay, thresholds, etc. After analyzing the results presented in the review, the authors reported that, as a result, the sensitivity and specificity for NT-pro-BNP in the diagnosis of hsPDA was 0.90 (95% CI, 0.79–0.96), and summary specificity was 0.84 (95% CI, 0.77–0.90) [42]. In the 11 included studies that evaluated NT-pro-BNP, there were eight in which measurements were performed on day 3 of life. The best predictive values for hsPDA were established for a threshold level of NT-pro-BNP in plasma of 11,395 pg/mL, 100% sensitivity with narrow confidence intervals (95% CI, 0.81–1.00) [43].

In two of the eight studies, sensitivity was lower than 0.7, with 42,285 (0.68, 95% CI 0.46–0.85) [44] and 10,253 (0.58 95% CI (0.33–0.8) used as a threshold [45].

In other studies, the sensitivity ranged from 0.85 to 0.95 (95% CI from 0.59 to 1.0) for NT-pro-BNP cut-off values of 5000 to 24,102 [46,47,48].

In comparison with the presented studies, the result of our study, which determined the threshold for NT-pro-BNP as lg3.9 pg/mL, shows one of the best combinations of high sensitivity (94.2%, CI 0.85 to 0.98) and specificity (83.7%, CI 0.71 to 0.92) and a narrow confidence interval. We used the obtained threshold value of NT-pro-BNP to create a model capable of dividing patients with extremely low body weight according to the degree of hemodynamic significance of PDA into two functional classes. The predictive model derived from discriminant analysis successfully classified 85.9% of participants with an AUC of 0.89.

The use of Apgar and Silverman–Anderson scores in our predictive model as predictors has its advantages and disadvantages. The advantages include the unification of decision making, and the possibility of quantitative assessment. The disadvantages are the uncertainty of the forecast time lag, the static nature of the forecast, dependence on the population, and the absence of standards. Additionally, a limitation of the study is that surgeons participating in the study were aware of NT-pro-BNP values in patients. Another limitation of our study was the small cohort of patients; however, the predictor correlation values seem reasonable. There was some selection bias in the study cohort: we included only premature infants for blood sampling and the decision to request an echocardiogram was based on the opinion of the attending physician. Strictly speaking, scales can be used if it is proven that their application reliably improves prognosis, compared to decisions made by a doctor without using scales. We believe that in conditions of limited resources, this provision is important, since it allows replacement of the standard echocardiographic assessment of hsPDA in newborns of VLBW on the third day of life by a combination of the following factors: NT-pro-BNP level more than lg3.9 pg/mL, Apgar score ≤ 6 points, Silverman–Anderson score (RSS) ≥ 5 points.

In the future, we plan to conduct a study in which we will compare our model with a group in which only NT-pro-BNP will be determined, the group in which only echocardiographic assessment will be used as an internal control.

Undoubtedly, therapeutic approaches to the problem of PDA closure continue to evolve, and much is being rethought and re-evaluated. We believe that future research should be as individualized as possible and we hope to make a small contribution to the benefit of patients.

## 5. Conclusions

The results of this study show that the accuracy of the clinical prognosis of hsPDA in premature infants weighing less than 1500 g can be significantly improved using affordable and inexpensive predictors that allow newborns to be classified according to hsPDA risk groups: NT-pro-BNP level more than lg3.9 pg/mL, Apgar score ≤ 6 points, and Silverman–Anderson score ≥ 5 points. Further research in this area will help to improve understanding of the problem of functioning PDA in children born very prematurely, and awareness of the fine line between norm and pathology determines the use of the most sparing therapeutic approaches in this group of patients.

## Figures and Tables

**Table 1:** Clinical characteristics of patients.

Characteristics	hsPDA	Non-hsPDA	*p*-Value
weight (g)	976 ± 287	1049 ± 261	0.078
gestation (weeks)	27.2 ± 2.62	27.9 ± 2.47	0.072
male gender	24 (52.2)	29 (54.7)	0.879
vaginal delivery	8 (17.4)	18 (33.9)	0.071
antenatal steroids	19 (41.3)	26 (49.0)	0.493
Apgar score (≤6)	12 (26.1)	5 (9.4)	0.021
Silverman–Anderson score (≥5)	33 (71.7)	2 (49.1)	0.022
NT-pro-BNP level (pg/mL)	18.967 ± 12.518	3448 ± 3926	0.001

Categorical data are shown as *n* (%) of each group. Continuous variables are shown as mean ± standard deviation.

**Table 2:** Echocardiographic parameters.

Parameters	hsPDA	Non-hsPDA
DA (mm)	2.78 ± 0.72	1.26 ± 0.66
DA ≥ 1.5 mm	45 (97.8)	22 (41.5)
LA/Ao ≥ 1.4 mm	34 (73.9)	2 (3.8)
Retrograde descending aortic flow ≥ 50% of antegrade blood flow	35 (76.1)	4 (7.5)

**Table 3:** Classification results *.

Actual Classification	Predicted Group Membership		Total
	Non-hsPDA	hsPDA	
non-hsPDA	47 (88.7)	6 (11.3)	53
hsPDA	8 (17.4)	38 (82.6)	46
Total	55	44	99

* There were 47 non-hsPDA (88.7%) and 38 hsPDA (82.6%) cases correctly classified (*n* = 99). The positive and negative predictive values were 84.0 and 86.0%, respectively.

## Data Availability

The Russian Ethics Review Authority only granted publication of aggregated data, which means that individual data cannot be shared.

## Supplementary Materials

The following are available online at https://www.mdpi.com/article/10.3390/jcm10163729/s1, Table S1: Variables groups used for discriminant analysis.
