# Supplementary material for: Discriminant Analysis of Main Prognostic Factors Associated with Hemodynamically Significant PDA: Apgar Score, Silverman–Anderson Score, and NT-Pro-BNP Level
Source: J Clin Med. 2021 Aug 22;10(16):3729. doi: 10.3390/jcm10163729 (PMC8397198; doi:10.3390/jcm10163729)
Supplement: Supplementary file 1 [file jcm-10-03729-s001.zip › jcm-1305992-supplementary.pdf]

Table S1. Variables groups used for discriminant analysis

Parameter group	Item
Age and gender	• Gender<br>• Gestation age
Anthropometry	• Weight<br>• Height
Clinical assessment of the cardiovascular and respiratory systems at birth	• Apgar 1<br>• Apgar 5<br>• Apgar 10<br>• Respiratory Severity Score
Baby therapy after birth	• Surfactant therapy (yes / no)<br>• Inotrope therapy (yes / no)
Respiratory support type	• DUOPAP (yes / no)<br>• NCPAP (yes / no)<br>• Mechanical ventilation (yes / no)
Laboratory data	• NT-pro-BNP<br>• Hemoglobin<br>• Platelets
ECHO-criteria	• Diameter DA≥ 1.5 mm<br>• LA/Ao≥ 1.4 – 2.0<br>• Retograde descending aortic flow ≥ 50% of antegrade blood flow
Data of instrumental research methods	• The presence of infiltrative shadows based on the results of radiography (yes/no)
Maternal history	• Mother's age<br>• Chronic gynecological diseases<br>• Endometriosis<br>• Chronic adnexitis
Obstetric history	• Pregnancy parity<br>• Birth parity<br>• Operative labor (yes / no)<br>• Number of medical abortions<br>• Number of miscarriages<br>• Medical supervision throughout pregnancy (yes / no)<br>• Threat of miscarriage<br>• Therapy during pregnancy (yes / no)<br>• Presence of preeclampsia during present pregnancy<br>• The presence of IVF in a real pregnancy<br>• The state of the placenta in childbirth
Somatic diseases of mother	• Diseases of the genitourinary system (yes / no)<br>• Diseases of the endocrine system (yes / no)<br>• Diseases of the cardiovascular system, including arterial hypertension (yes / no)<br>• Chronic nicotine intoxication (yes / no)<br>• Chronic alcohol intoxication (yes / no)
